# 

**Published:** 1889-09

**Authors:** 


CONTENTS

Vol. 36 No. 9.

| | PAGE |
|---|---|
| Looking Forward | 193 |
| Health Without Medicine | 197 |
| Pernicious Swindles | 200 |
| A View of the Marriage Question | 201 |
| Weak Hearts | 201 |
| Why we are Right Handed | 202 |
| How Homes are Wrecked | 203 |
| The Rattlesnake | 204 |
| Liquorice Cultivation | 205 |
| How Glucose is Made | 206 |
| For Dandruff | 207 |
| The Microbes in Milk and Water | 207 |
| Hair Dressing | 208 |
| Coffee and Typhoid Fever | 208 |
| Convulsions in Children | 208 |
| A Skeptic's Testimony | 209 |
| To Cure Drunkenness | 212 |
| Household | 213 |
| Miscellaneous | 214 |
| Book Notices | 216 |

INDEX TO ADVERTISEMENTS OF HALF A PAGE AND OVER.

| | PAGE. |
|---|---|
| The Consumers' Supply Ass'n. | I |
| Celluloid | II |
| Hollow Suppositories | III |
| E. C. Morris & Co | IV |
| Lactopeptine | V |
| An Unprecedented Offer | VI |
| Revised Premium List | VIII |
| The Herb Cure Co | X |
| Ladies' Home Companion | XI |

